# Polycyclic aromatic hydrocarbons in the snow cover of the northern city agglomeration

**DOI:** 10.1038/s41598-021-98386-x

**Published:** 2021-09-24

**Authors:** A. Yu. Kozhevnikov, D. I. Falev, S. A. Sypalov, I. S. Kozhevnikova, D. S. Kosyakov

**Affiliations:** Laboratory of Environmental Analytical Chemistry, Core Facility Center “Arktika”, Northern (Arctic) Federal University, Arkhangelsk, Russia

**Keywords:** Environmental chemistry, Environmental chemistry

## Abstract

Sixteen priority polycyclic aromatic hydrocarbons (PAHs) were qualitatively and quantitatively assessed by high-performance liquid chromatography with fluorescence detection in snow samples collected at 46 sites of Arkhangelsk as a world’s largest city above 64 degrees north latitude. The average, maximum and minimum PAH concentrations in snow were 168, 665, and 16 ng/kg, respectively. The average toxic equivalent value in benzo(a)pyrene units was 3.6 ng/kg, which is three-fold lower than the established maximum permissible concentration and considered an evidence of a low/moderate level of snow pollution with PAHs. The pollution origin was assessed using specific markers based on PAHs ratios in the studied samples. The pyrogenic sources of PAH emission were predominate, whereas the significant contributions from both transport and solid fuel combustion were observed. Benzo(a)pyrene concentrations are highly correlated with the levels of other PAHs with higher molecular weights.

## Introduction

Polycyclic aromatic hydrocarbons (PAHs) are persistent hazardous organic compounds that enter the environment during incomplete combustion of biomass (including peat fires) and fossil fuels, food cooking, and the leakage of crude oil, mineral oils and bitumen during their industrial use and transportation^1–4^. For this reason, PAHs are typical contaminants of the atmosphere and soils of urban areas^5–11^. Due to high mutagenic, teratogenic, and carcinogenic activity^12^, these compounds are included in the group of priority pollutants, which are most dangerous for human health and natural ecosystems. In addition to the three mentioned hazardous properties, exposure of animals and humans to PAHs by inhalation, direct contact and ingestion can cause a number of other adverse effects, such as reproductive- and endocrine-disrupting action, neurotoxicity, and oxidative stress^13^. As a rule, toxicity of PAHs increases with the number of aromatic rings in their structure. An important factor that enhances the toxic effect of PAHs is their high ability to accumulate in living organisms^14^.

Despite the fact that hundreds of PAHs as well as their numerous derivatives (for example, oxygenates) are currently known, the existing regulations provide for the analysis of a limited number of the highest priority compounds of this group, selected on the basis of their prevalence, persistence and toxicity. The widest range of such priority PAHs is listed by the United States Environmental Protection Agency (US EPA) and includes 16 compounds containing from 2 to 6 rings in their structure: naphthalene (N), acenaftilene (AN), acenaphthene (ACE), fluorene (F), phenanthrene (PHE), anthracene (ANT), fluoranthrene (FLT), pyrene (PYR), benz(a)anthracene (BaA), chrysene (CHR), benzo(b)fluoranthene (BbF), benzo(k)fluoranthene (BkF), benzo(a)pyrene (BaP), dibenz(a,h)anthracene (dBahA), benzo(g,h,i)perylene (BghiP), indeno[1,2,3-c,d]pyrene (IND)^15^. It should be noted that in some countries, including Russia, only BaP, which has the highest carcinogenic activity, is controlled according to official regulatory requirements.

Special attention should be paid to the control of PAHs in the Arctic region, the natural ecosystems of which are extremely fragile, while the cold climate promotes the accumulation and long-term preservation of pollutants. Despite the absence of large-scale industry, PAHs were found in the atmospheric air of high latitudes at significant levels reaching 1 µg/m^3^ (sum concentration)^16,17^. It is worth noting that despite the substantial reduction in PAH global emissions during the last three decades no significant decline of the levels of major PAHs were observed in Arctic^17,18^. On the other hand, the recent studies in Franz Joseph Land archipelago showed an extremely low PAH level below detection limit of gas chromatography—high-resolution mass spectrometry technique^19^. Along with long-range transport, large urban agglomerations in the arctic and subarctic regions can act as serious sources of PAHs in high latitudes; therefore, the control of PAH contamination on their territory is an urgent task^20–22^. A feature of the northern territories is the presence of snow cover throughout most of the year. PAHs drop out of the atmosphere during snowfall or condense on the snow surface (cold finger effect^23^ accumulating in seasonal snow cover []. Thus, PAH levels in fresh snow and snow cover adequately reflects the pollution of atmospheric air^22^. On the other hand, seasonal snow thawing results in the release of large amounts of PAHs into the Arctic Ocean basin and atmosphere^24,25^. From this point of view, the study of PAH contamination of urban snow is of particular interest^26,27^. In this regard, it is worth noting that snow is considered preferred analytical matrix for analysis of atmospheric pollutants due to its availability, easy sampling procedure and minimum matrix interferences. Recently, it was successfully used for targeted determination and non-targeted screening of wide range of volatile and semi-volatile airborne pollutants, including a number of PAHs, in Arctic^28^.

The literature data on the pollution of northern cities with PAHs are extremely scarce so far. In this regard, of greatest interest is the recent paper by Vijayan^29^ reporting the levels of 16 priority PAHs in the snow of two Swedish cities, Luleå and Umeå. The determined sum concentrations were 2.7 and 9.6 μg/kg, respectively, whereas individual PAH concentrations varied from 0.06 to 0.58 μg/kg in Luleå and 0.08 to 2.31 μg/kg in Umeå. Most of the available publications are mainly devoted to the situation in large urban agglomerations of the temperate climatic zone with much higher anthropogenic load and pollution levels. Surprisingly, the maximum total PAH content in the snow of the Moscow (ring road zone) in 2012^30^ was close to that in Luleå^31^. Lower PAH contents (0.04–0.7 μg/kg) in snow were measured in Khabarovsk city^13^. Much higher levels were observed in Irkutsk region of Siberia (up to 135 μg/L^31^) and northern China^27^ which can be explained by the widespread use of coal as a fuel. The total PAH content in the fresh snow and snow cover in Changchun city ranged from 27 to 37 and from 40 to 106 μg/kg, respectively^22^. Snow from Harbin contained 16 PAHs with contents in the range of 0.3–2550 μg/kg (~ 4 orders of magnitude)^26^. The most abundant compounds were PYR (17%), followed by PHE (15%), N (14%), and FLT (10%).

The aim of the present study was to expand the knowledge about priority PAHs levels in the snow cover of northern urban agglomerations using the example of Arkhangelsk as the world's largest city above 64 degrees north latitude.

Arkhangelsk, a city in the north of the European Russia with a population of about 350,000 and a large number of vehicles (> 144,000) is known as one of the industrial centers of Russia with developed pulp and paper industry, shipbuilding, power generation, transport and a large port. The annual emission of all monitored atmospheric pollutants from the territory of Arkhangelsk region is about 250,000 tons, 59% of which originate from stationary sources and 41% from transport. Average concentrations of pollutants in the atmospheric air are usually below sanitary standards^32^.

## Results and discussion

**Figure 1:**
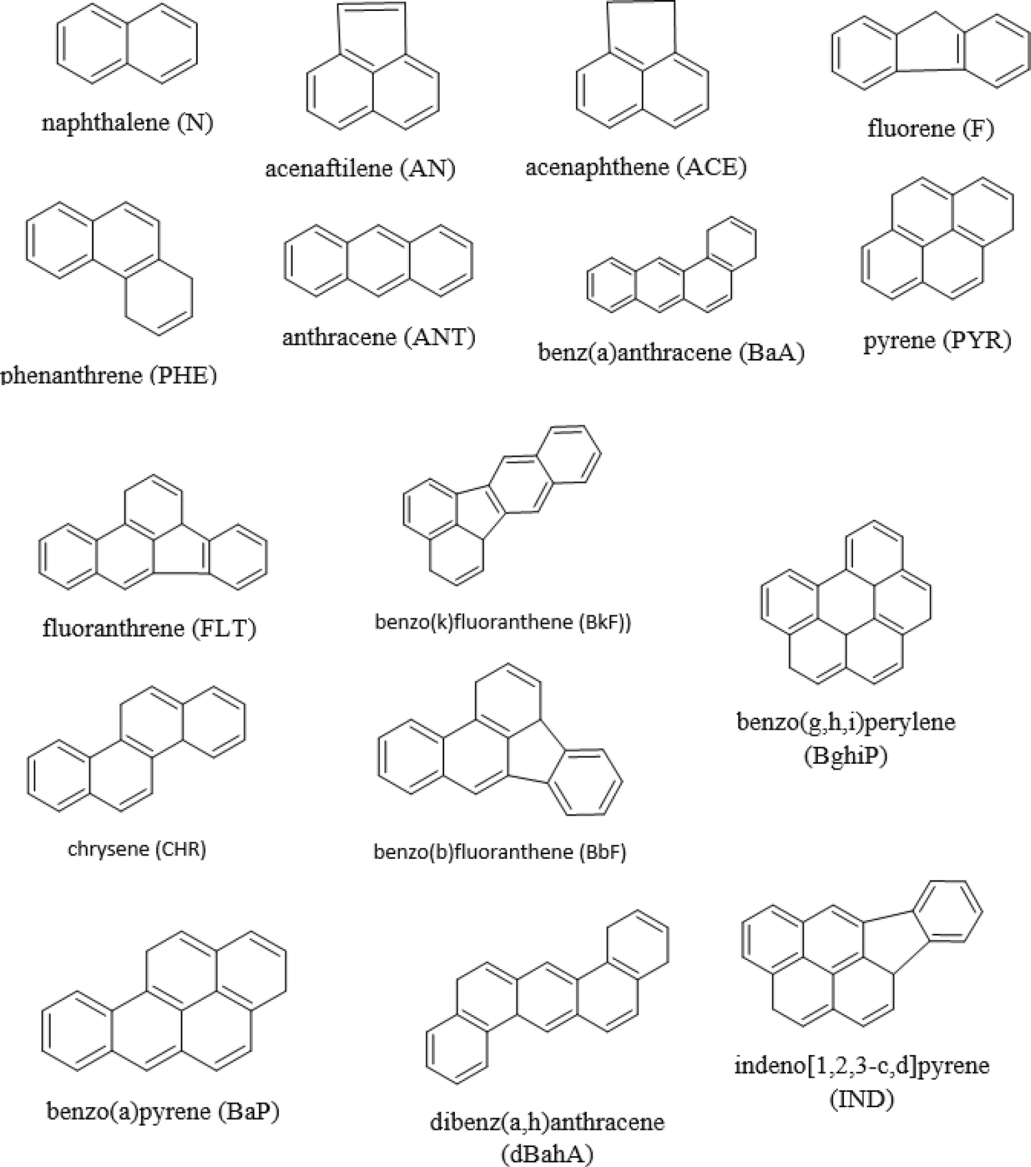
Structural formulas of priority PAHs.

**Figure 2:**
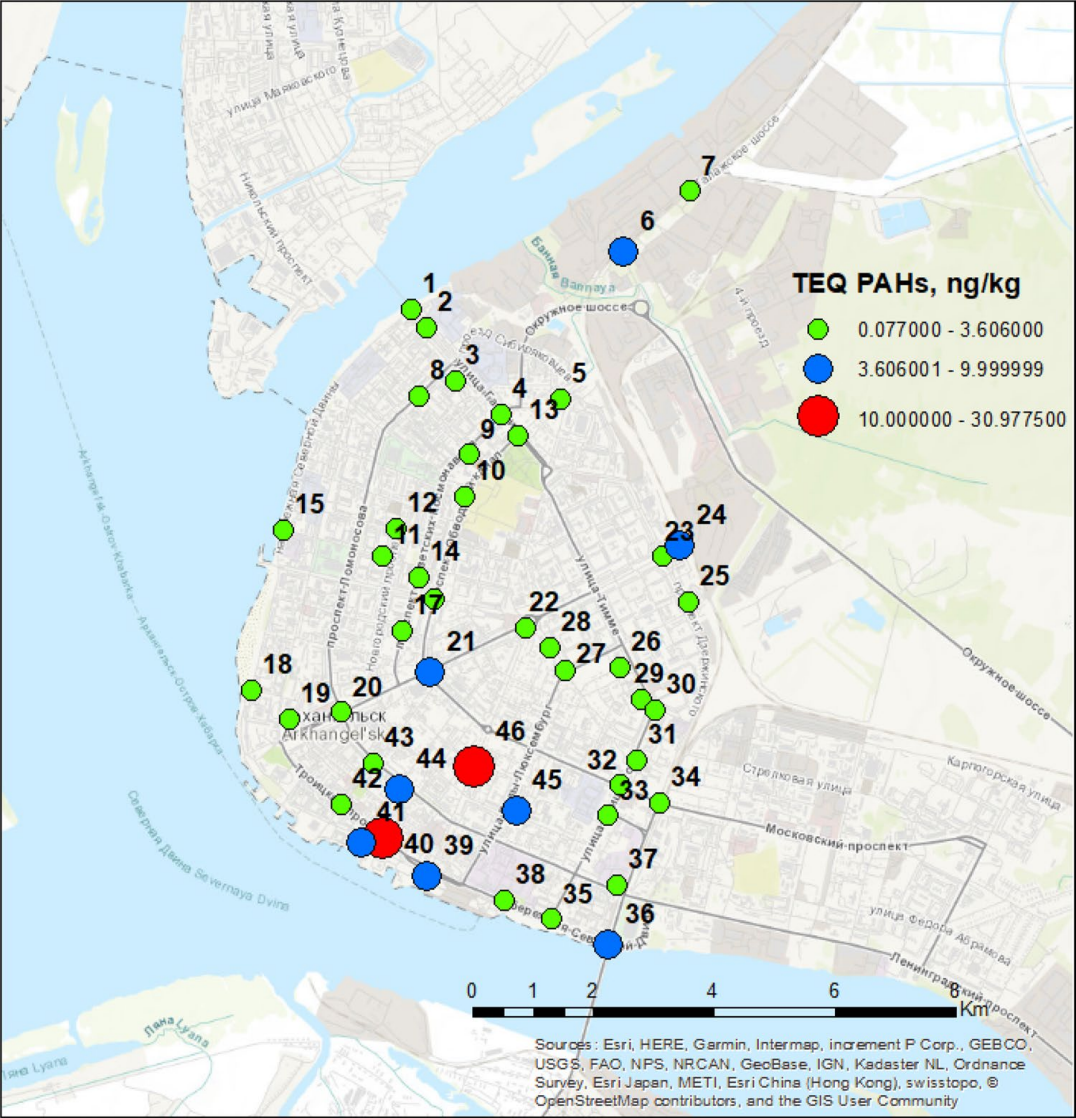
Snow sampling points and the toxic equivalency value. The map was created using the ArcGIS ver. 10.4.1 for desktop (https://desktop.arcgis.com/en/).

**Figure 3:**
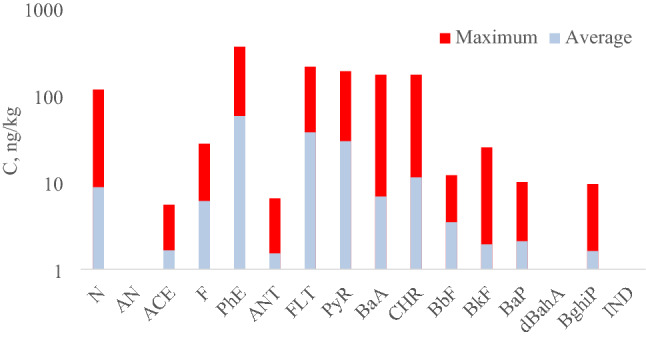
The distribution of priority PAHs for individual components.

Sixteen priority PAHs (Fig. 1) were selected as target analytes in accordance with US EPA guidelines (see “Introduction” section). For the study, snow samples were taken from 46 points on the territory of Arkhangelsk (Fig. 2). The results of their analysis by high performance liquid chromatography with fluorescence detection (HPLC-FLD) are presented in the Supplementary material (Table S1). The average, maximum and minimum values of the total PAHs content (Σ_16_ PAH) in the studied snow samples were 0.17, 0.65, and 0.016 μg/kg, respectively. The distribution of priority PAHs for individual components was very uneven (Fig. 3). In general, 6 analytes predominated in the studied samples: N, PHE, FLT, PYR, BaA, CHR. Their averaged contents were in the range of 0.005–0.050 μg/kg, whereas maximum values exceeded 0.1 μg/kg. ACE, F, ANT, BbF, BkF, BaP, and BghiP were found in noticeable quantities in some samples. The remaining three analytes (AN, dBahA, IND) were not detected in any sample.

The maximum level of Σ_16_ PAH was found at sampling point No. 36 in the area of the bridge over the Northern Dvina river which is characterized by high road traffic, the presence of traffic jams and the passage of railway tracks. The minimum levels of Σ_16_ PAH (0.016 μg/kg) were observed in areas away from major transport routes.

The comparison of the obtained PAH concentrations with those measured in Moscow snow^33^ indicates an order of magnitude lower level of pollution in Arkhangelsk. Moreover, even the maximum measured concentrations of PAHs were inferior to the average levels in Swedish cities similar to Arkhangelsk in terms of climatic conditions^29^. This can be explained not only by the difference in the structure of industry and transport, but also by the difference in the snow sampling season—samples studied in^6^ were collected in mid-March, just before the final snowmelt and thus accumulated more pollutants. Another explanation is based on the fact that Arkhangelsk has centralized power and heat generation source (central power plant) using natural gas as a fuel and supplying more than 90% of city’s population and industry. The wood and coal-based heat generation is used only in suburban areas far from sampling sites. This dramatically reduces the air and snow contamination with PAHs.

Due to differences in the toxicity of individual PAHs, an estimation of the snow pollution based on the total content of these compounds cannot be considered acceptable. In such a situation, an approach based on the use of toxic equivalents of individual compounds can be used. Since BaP is one of the most toxic, carcinogenic and persistent compounds among PAHs, and its maximum permissible concentration (MPC) is legislatively established in Russia (10 ng/L in water), this analyte was used as a reference substance (BaP concentration units) for expressing the toxic equivalence (TEQ) of the studied samples. The obtained results (Fig. 1) demonstrate that MPC was exceeded only at two points (marked in red) located near the administrative center of the city. The average TEQ value (3.6 ng/kg) was three-fold lower than MPC and was exceeded at 7 of 46 sampling points (marked in blue) which is an evidence of a low/moderate level of air pollution with PAHs.

It is worth noting that BaP concentrations are highly correlated with the levels of other PAHs with higher molecular weights (ANT, AN, FLT, PyR, BaA, CHR, BbF, BkF, BghiP) whereas there are no good correlations of BaP with N, ACE, and PHE (Table 1). This can be considered an evidence of different origins of these two groups of analytes. The more substantiated conclusions about the sources of PAHs can be made based on the analysis of individual analytes (or their groups) ratios^10^ (Table 2).

**Table 1:** Pearson's correlation of individual PAH concentrations in the snow samples.

N	1												
ACE	0.108	1											
F	0.277	0.37	1										
PHE	0.069	0.095	0.638**	1									
ANT	0.083	0.285	0.661**	0.491**	1								
FLT	0.138	0.472*	0.657**	0.552**	0.741**	1							
PyR	0.15	0.381	0.525**	0.489**	0.717**	0.933**	1						
BaA	0.025	0.294	0.16	0.003	0.439**	0.207	0.165	1					
CHR	0.031	0.334	0.284	0.119	0.542**	0.348*	0.300*	0.985**	1				
BbF	0.057	0.562**	0.457**	0.258	0.721**	0.685**	0.621**	0.582**	0.646**	1			
BkF	0	0.387	0.167	0.009	0.27	0.275	0.253	0.287	0.28	0.430**	1		
BaP	0.101	0.374	0.400**	0.241	0.792**	0.559**	0.552**	0.660**	0.718**	0.881**	0.457**	1	
BghiP	0.135	0.359	0.258	0.117	0.633**	0.403**	0.398**	0.776**	0.819**	0.822**	0.407**	0.935**	1
	N	ACE	F	PHE	ANT	FLT	PyR	BaA	CHR	BbF	BkF	BaP	BghiP

*Correlation is significant at the 0.05 level (two-sided).

**Correlation is significant at the 0.01 level (two-sided).

**Table 2:** The ratios PAH concentrations in the snow samples collected at different sampling points.

Point N	(PYR + BaP)/ (PHE + CHR)	(PYR + BaP + BghiP)/ (PHE + CHR)	ANT/ (ANT + PHE)	FLT/ (FLT + PYR)	BaA/ (BaA + CHR)	HMW/LMW
1	0.2	0.2	0.0	0.6	0.1	0.26
2	0.8	0.9	0.1	0.7	0.1	0.46
3	0.4	0.4	0.0	0.6	0.2	0.32
4	**4.9**	5.0	0.1	0.2	0.2	2.12
5	0.4	0.4	0.0	0.6	0.2	0.49
6	0.4	0.4	0.0	0.5	0.1	0.40
7	0.3	0.3	0.0	0.5	0.1	0.30
8	0.5	0.6	0.0	0.6	0.1	0.39
9	0.4	0.4	0.0	0.6	0.2	0.38
10	0.6	0.7	0.0	0.6	0.1	0.58
11	0.2	0.2	0.1	0.6	0.0	0.93
12	0.7	0.8	0.1	0.6	0.2	0.43
13	0.1	0.1	0.0	0.6	0.2	0.20
14	0.0	0.0	0.0	0.6	0.2	0.07
15	0.7	0.8	0.0	0.6	0.2	0.70
16	0.6	0.6	0.0	0.6	0.0	0.40
17	0.8	0.8	0.0	0.6	0.2	0.55
18	**1.1**	1.2	0.1	0.5	0.1	0.74
19	0.5	0.5	0.0	0.6	0.2	0.38
20	0.5	0.5	0.0	0.6	0.1	0.37
21	0.6	0.7	0.0	0.5	0.3	0.65
22	0.5	0.5	0.1	0.6	0.0	0.39
23	**1.1**	1.2	0.1	0.5	0.3	0.65
24	0.5	0.5	0.0	0.6	0.3	0.45
25	0.4	0.4	0.0	0.6	0.0	0.33
26	0.8	0.8	0.1	0.6	0.0	0.53
27	0.5	0.5	0.0	0.6	0.4	0.64
28	0.6	0.6	0.1	0.6	0.4	0.44
29	0.2	0.2	0.0	0.6	0.0	0.18
30	0.2	0.2	0.0	0.6	0.0	0.22
31	0.8	0.9	0.0	0.6	0.2	0.61
32	0.3	0.3	0.0	0.6	0.2	0.32
33	0.8	0.8	0.0	0.5	0.2	0.28
34	0.3	0.3	0.0	0.7	0.2	0.30
35	0.5	0.5	0.0	0.6	0.2	0.41
36	0.2	0.2	0.0	0.6	0.2	0.25
37	0.5	0.6	0.0	0.6	0.0	0.39
38	0.4	0.4	0.1	0.6	0.0	0.36
39	0.9	1.0	0.1	0.6	0.2	0.74
40	**1.3**	1.4	0.1	0.5	0.2	0.84
41	0.9	0.9	0.0	0.5	0.3	0.55
42	0.7	0.7	0.0	0.6	0.2	0.48
43	0.5	0.5	0.0	0.5	0.2	0.42
44	**1.1**	1.2	0.1	0.6	0.5	0.94
45	0.6	0.6	0.1	0.6	0.1	0.47
46	0.3	0.3	0.1	0.6	0.5	2.93

The (PYR + BaP)/(PHE + CHR) ratio was used as a specific marker to establish the relationship between PAHs of technogenic and natural origins. The ratio values > 1 indicate the prevalence of technogenic sources^10^. Analyzing the data obtained, one can see that only at four points there is an excess of this parameter, in two of them, by only 10%. The value significantly exceeds 1 only at one point (No. 4) where there is also a very large traffic of vehicles. The average value of this ratio was 0.5, which indicates that the urban agglomeration of Arkhangelsk is not contaminated only with PAHs of technogenic origin. It is worth noting that it is difficult to discriminate the combustion of wood biomass for energy production and natural sources of PAHs such as fires.

The values of the ratio (PYR + BaP + BghiP) / (PHE + CHR). These values also showed an excess of 1 only at the aforementioned points, on average this parameter was 0.5 units. at the point where this ratio exceeds 1, this indicates the anthropogenic origin of pollution^10^.

The ANT/(ANT + PHE) ratio uses the PAHs with molecular weights of 178 Da and allows distinguishing between petroleum (value < 0.1) and combustion (> 0.1) sources^10^. For the majority of sample points, the measured values were close to zero indicating the oil pollution. At 13 sites the value of 0.1 was achieved. This boundary level indicates that the observed PAHs are of mixed oil origin and origin from combustion, however, this ratio can be applied only with sufficient restrictions and using this parameter alone it is impossible to reliably assert the origin of PAHs.

The FLT/(FLT + PYR) ratio^34,35^ serves as another criterion to discriminate combustion and non-combustion origins of PAHs. The obtained average value of 0.6 (> 0.4–0.5) can be considered a reliable evidence for combustion as a main source of PAHs in snow and corresponds to the use of coal/wood as a fuel. This is in a good agreement with the fact that coal, wood and other types of biomass are widely used in Arkhangelsk for generating heat.

The BaA/(BaA + Chr) ratio provides a more definitive indicator of vehicle emissions than any described above^29^. Since the liquid fuel combustion produces BaA more efficiently than solid fuel combustion, the high values (> 0.35) of BaA/(BaA + Chr) testify to the greatest contribution of transport to PAH pollution. In our case, this ratio varied from 0 to 0.5 and typically was below 0.35. (average value is 0.2). This is an evidence for the mixed source of PAHs and significant role of wood/coal combustion. This additionally proves the conclusion made on the basis of FLT/(FLT + PYR) ratio.

High molecular weight (HMW) / low molecular weight (LMW) PAHs ratio did not exceed 0.5, which once more clearly indicates pyrolytic origin of pollution^36^. Only at 2 points (No. 4 and 46) this ratio was more than 1.

Summarizing the criterions described above one can conclude that PAHs in Arkhangelsk snow originate predominantly from combustion processes with significant contributions from both transport and solid fuel combustion.

## Material and methods

Analytical standards for quantification purposes were purchased from Sigma-Aldrich (Steinheim, Germany) as a certified reference material containing 16 priority PAHs in methanol with concentrations of 10 µg/mL. HPLC-hypergradient grade acetonitrile (Cryochrom, Moscow, Russia) was used for the preparation of sample and standard solutions and as a component of mobile phase in chromatographic analysis. HPLC grade hexane (Cryochrom, Moscow Russia) was used in a sample preparation procedure for analytes extraction. High purity "type I" water was obtained using Milli-Q system (Millipore, Molsheim, France).

The snow samples were taken from the most and least loaded intersections of the Arkhangelsk city within 4 h from 10 am to 2 pm on January 13, 2020. The air temperature and humidity were − 13 °C and 66%, respectively. There were no precipitations during 5 days before sampling whereas the monthly amount of precipitation was 22–48 mm, which corresponds to 63–97% of the normal value. The average snow depth was 15 cm. Sampling was carried out using a special metal core with a diameter of 10 cm. The surface layer of snow was not sampled. The collected snow samples were placed in 1-L dark glass bottles was thawed at room temperature. Extraction of PAHs was carried out with 5 mL of hexane for 30 min with vigorous stirring. The hexane extract was separated from water, poured into a glass flask and evaporated at a temperature of 60 °C under pure air flow to a volume of 0.5–1 mL. The remainder of the extract was transferred quantitatively into a 1.5-mL vial and left until the hexane was completely volatilized. At the end of the evaporation, 0.5 mL of acetonitrile was added to the vial. Thus, the concentration factor was 2000. The prepared extracts were filtered using syringe nylon membrane filters with a pore size of 0.2 μm and subjected to the chromatographic analysis on the same day.

Analysis of PAHs was carried out by high-performance liquid chromatography with fluorescence detection (HPLC-FLD) using an Agilent 1260 HPLC system (Agilent, Santa Clara, USA) consisted of a binary chromatographic pump, autosampler, column oven, diode array spectrophotometric and fluorescence detectors. Chromatographic separation was achieved on a LiChrospher PAH column (Agilent, Santa Clara, USA), 250 × 3 mm, particle size 5 μm at a temperature 20 °C. The injection volume was 20 μL. A mixture of water (A) with acetonitrile (B) was used as a mobile phase at a flow rate of 0.56 mL/min. A gradient elution was used with the following program: 0–3 min: 50% B, 10 min–100% B. The total analysis time was 28 min. Detection was carried out with time programming of the excitation (λ_ex_) and emission (λ_em_) wavelengths: 0–12 min: λ_ex_ = 280 nm, λ_em_ = 340 nm; 12.0–12.8 min: λ_ex_ = 292 nm, λ_em_ = 336 nm; 12.8–13.2 min: λ_ex_ = 253 nm, λ_em_ = 402 nm; 13.2–13.8 min: λ_ex_ = 360 nm, λ_em_ = 460 nm; 13.8–16.1 min: λ_ex_ = 305 nm, λ_em_ = 430 nm; 16.1–17.0 min: λ_ex_ = 268 nm, λ_em_ = 383 nm; 17–28 min: λ_ex_ = 305 nm, λ_em_ = 430 nm. The identification of the compounds was confirmed by UV absorption spectra.

Calibration solutions with the concentration of each compound in the range of 0.5–0.001 µg/mL were prepared by sequential dilution of the standard solution with acetonitrile (10 mg/L each) immediately before the experiment. All calibration dependences in the studied range were linear and the correlation coefficient was > 0.999. An example of a chromatogram of one of the investigated snow samples (Supplementary material, Fig. S1) demonstrates good separation of all analytes with the absence of noticeable matrix interferences.

The toxic equivalence at each sampling point was calculated using the formula

$$ TEQ = \sum \left( {C_{i} *TEF_{i} } \right) $$

taking into account the contribution of each PAH to the total toxicity of the most dangerous compound BaP^37^. The contribution of other PAHs (C_i_) was calculated according to the coefficients: PHE (0.001), ANT (0.01), AN (0.02), BaA (0.1), FLT (0.001), PYR (0.001), CHR (0.01), BbF (0.1), BkF (0.1), BaP (1), dBahA(0.01), BghiP (0.01)^10^.

Statistical calculations were performed using software package IBM SPSS STATISTICS Ver. 23. We have carried out a static regression analysis (calculation of the equation of the relationship between two values); paired correlation coefficients (*r*) at a certain level of significance (*p* ≤ 0.05).

## Supplementary Information

Supplementary Information.
