# Supplementary material for: Polycyclic aromatic hydrocarbons in the snow cover of the northern city agglomeration
Source: Sci Rep. 2021 Sep 24;11:19074. doi: 10.1038/s41598-021-98386-x (PMC8463559; doi:10.1038/s41598-021-98386-x)
Supplement: Supplementary file 1 — Supplementary Information. [file 41598_2021_98386_MOESM1_ESM.docx]

**Supplementary Information**

**Polycyclic aromatic hydrocarbons in the snow cover of the northern city agglomeration**

A.Yu. Kozhevnikov^*^, D. I. Falev, S.A. Sypalov, I.S. Kozhevnikova, D.S. Kosyakov

*Corresponding Autor: [akozhevnikov@mail.ru](mailto:akozhevnikov@mail.ru)

Laboratory of Environmental Analytical Chemistry, Core Facility Center “Arktika”, Northern (Arctic) Federal University, Arkhangelsk, Russia

Table S1. Concentrations PAHs in snow at sampling points, µg/kg

|  | naphthalene | acenaftilene | acenaphthene | fluorene | phenanthrene | anthracene | fluoranthene | pyrene | benz(a)anthracene | chrysene | benzo(b)fluoranthene | benzo(k)fluoranthene | benzo(a)pyrene | dibenz(a,h)anthracene | benzo(g,h,i)perylene | indeno[1,2,3-c,d]pyrene |
| --- | --- | --- | --- | --- | --- | --- | --- | --- | --- | --- | --- | --- | --- | --- | --- | --- |
| sampling point | N | AN | ACE | F | PhE | ANT | FLT | PyR | BaA | CHR | BbF | BkF | BaP | dBahA | BghiP | IND |
| 1 | 0.0055 | - | - | 0.0040 | 0.0935 | 0.0005 | 0.0250 | 0.0180 | 0.0010 | 0.0080 | 0.0035 | 0.0010 | 0.0015 | - | 0.0010 | - |
| 2 | 0.0035 | - | 0.0005 | 0.0065 | 0.0185 | 0.0010 | 0.0425 | 0.0205 | 0.0010 | 0.0065 | 0.0025 | 0.0010 | 0.0005 | - | 0.0010 | - |
| 3 | 0.0145 | - | 0.0055 | 0.0155 | 0.1205 | 0.0015 | 0.0840 | 0.0475 | 0.0035 | 0.0145 | 0.0060 | 0.0020 | 0.0020 | - | 0.0015 | - |
| 4 | 0.0120 | - | - | 0.0020 | 0.0120 | 0.0010 | 0.0135 | 0.0750 | 0.0010 | 0.0035 | 0.0025 | 0.0010 | 0.0015 | - | 0.0015 | - |
| 5 | 0.0110 | - | - | 0.0025 | 0.0190 | 0.0005 | 0.0125 | 0.0080 | 0.0010 | 0.0055 | 0.0035 | 0.0015 | 0.0015 | - | 0.0015 | - |
| 6 | 0.0225 | - | 0.0010 | 0.0095 | 0.1750 | 0.0020 | 0.0790 | 0.0715 | 0.0030 | 0.0230 | 0.0060 | 0.0025 | 0.0045 | - | 0.0040 | - |
| 7 | 0.0165 | - | - | 0.0065 | 0.0555 | 0.0015 | 0.0215 | 0.0185 | 0.0010 | 0.0065 | 0.0015 | 0.0010 | 0.0010 | - | 0.0005 | - |
| 8 | 0.0090 | - | - | 0.0090 | 0.0275 | 0.0005 | 0.0290 | 0.0165 | 0.0010 | 0.0060 | 0.0025 | 0.0010 | 0.0015 | - | 0.0010 | - |
| 9 | 0.0105 | - | 0.0005 | 0.0070 | 0.0505 | 0.0020 | 0.0340 | 0.0230 | 0.0020 | 0.0075 | 0.0035 | 0.0010 | 0.0015 | - | 0.0010 | - |
| 10 | 0.0100 | - | - | 0.0045 | 0.0170 | 0.0005 | 0.0195 | 0.0140 | 0.0010 | 0.0080 | 0.0030 | 0.0010 | 0.0020 | - | 0.0010 | - |
| 11 | 0.0050 | - | - | 0.0025 | 0.0080 | 0.0005 | 0.0070 | 0.0050 | 0.0005 | 0.0150 | 0.0005 | - | 0.0005 | - | - | - |
| 12 | 0.0055 | - | 0.0005 | 0.0025 | 0.0135 | 0.0010 | 0.0145 | 0.0110 | 0.0005 | 0.0020 | 0.0010 | 0.0005 | 0.0005 | - | 0.0005 | - |
| 13 | 0.0035 | - | 0.0005 | 0.0030 | 0.0530 | 0.0010 | 0.0085 | 0.0060 | 0.0010 | 0.0045 | 0.0005 | 0.0005 | 0.0010 | - | 0.0005 | - |
| 14 | 0.0010 | - | 0.0005 | 0.0040 | 0.3200 | 0.0010 | 0.0210 | 0.0115 | 0.0010 | 0.0045 | 0.0030 | 0.0010 | 0.0015 | - | 0.0010 | - |
| 15 | 0.0020 | - | - | 0.0015 | 0.0150 | 0.0005 | 0.0180 | 0.0125 | 0.0010 | 0.0045 | 0.0030 | 0.0010 | 0.0020 | - | 0.0020 | - |
| 16 | 0.0040 | - | 0.0015 | 0.0045 | 0.0245 | 0.0005 | 0.0270 | 0.0160 | - | 0.0040 | 0.0025 | 0.0005 | 0.0005 | - | 0.0010 | - |
| 17 | 0.0035 | - | - | 0.0055 | 0.0320 | 0.0010 | 0.0420 | 0.0295 | 0.0020 | 0.0075 | 0.0045 | 0.0010 | 0.0010 | - | 0.0010 | - |
| 18 | 0.0015 | - | - | 0.0020 | 0.0085 | 0.0005 | 0.0140 | 0.0120 | 0.0005 | 0.0030 | 0.0020 | 0.0005 | 0.0010 | - | 0.0005 | - |
| 19 | 0.0035 | - | - | 0.0055 | 0.0285 | 0.0005 | 0.0210 | 0.0145 | 0.0010 | 0.0040 | 0.0015 | 0.0005 | 0.0005 | - | 0.0005 | - |
| 20 | 0.0045 | - | 0.0015 | 0.0060 | 0.0210 | 0.0010 | 0.0160 | 0.0120 | 0.0005 | 0.0035 | 0.0010 | 0.0005 | 0.0005 | - | 0.0005 | - |
| 21 | 0.0040 | - | 0.0005 | 0.0055 | 0.0400 | 0.0015 | 0.0305 | 0.0270 | 0.0045 | 0.0085 | 0.0050 | 0.0015 | 0.0040 | - | 0.0025 | - |
| 22 | 0.0015 | - | - | 0.0005 | 0.0045 | 0.0005 | 0.0045 | 0.0030 | - | 0.0010 | 0.0005 | - | - | - | - | - |
| 23 | 0.0075 | - | 0.0010 | 0.0025 | 0.0190 | 0.0010 | 0.0260 | 0.0255 | 0.0015 | 0.0045 | 0.0020 | 0.0010 | 0.0015 | - | 0.0010 | - |
| 24 | 0.0075 | - | 0.0005 | 0.0170 | 0.0720 | 0.0035 | 0.0580 | 0.0380 | 0.0070 | 0.0145 | 0.0060 | 0.0025 | 0.0030 | - | 0.0005 | - |
| 25 | 0.0050 | - | 0.0020 | 0.0045 | 0.0215 | 0.0005 | 0.0145 | 0.0100 | - | 0.0030 | 0.0015 | 0.0005 | 0.0005 | - | 0.0005 | - |
| 26 | 0.0050 | - | - | 0.0050 | 0.0245 | 0.0020 | 0.0290 | 0.0230 | - | 0.0055 | 0.0035 | 0.0010 | 0.0015 | - | 0.0005 | - |
| 27 | 0.0005 | - | 0.0005 | 0.0030 | 0.0220 | 0.0005 | 0.0175 | 0.0120 | 0.0040 | 0.0065 | 0.0020 | 0.0010 | 0.0015 | - | 0.0010 | - |
| 28 | 0.0015 | - | - | 0.0010 | 0.0085 | 0.0005 | 0.0090 | 0.0050 | 0.0010 | 0.0015 | 0.0005 | 0.0005 | 0.0005 | - | - | - |
| 29 | 0.0035 | - | - | 0.0035 | 0.0945 | 0.0005 | 0.0205 | 0.0140 | - | 0.0045 | 0.0020 | 0.0005 | 0.0010 | - | 0.0005 | - |
| 30 | 0.0040 | - | 0.0005 | 0.0035 | 0.0455 | 0.0010 | 0.0160 | 0.0105 | - | 0.0025 | 0.0010 | 0.0005 | 0.0005 | - | 0.0005 | - |
| 31 | 0.0040 | - | - | 0.0060 | 0.0255 | 0.0010 | 0.0400 | 0.0260 | 0.0020 | 0.0085 | 0.0050 | 0.0015 | 0.0020 | - | 0.0020 | - |
| 32 | 0.0050 | - | - | 0.0055 | 0.0490 | 0.0010 | 0.0240 | 0.0160 | 0.0010 | 0.0045 | 0.0025 | 0.0010 | 0.0015 | - | 0.0005 | - |
| 33 | 0.1150 | - | 0.0005 | 0.0105 | 0.0425 | 0.0015 | 0.0485 | 0.0400 | 0.0020 | 0.0105 | 0.0030 | 0.0015 | 0.0025 | - | 0.0025 | - |
| 34 | 0.0065 | - | 0.0040 | 0.0120 | 0.0810 | 0.0005 | 0.0425 | 0.0225 | 0.0025 | 0.0080 | 0.0045 | 0.0015 | 0.0025 | - | 0.0025 | - |
| 35 | 0.0045 | - | - | 0.0030 | 0.0370 | 0.0005 | 0.0245 | 0.0175 | 0.0010 | 0.0050 | 0.0020 | 0.0005 | 0.0015 | - | 0.0010 | - |
| 36 | 0.0175 | - | 0.0015 | 0.0275 | 0.3560 | 0.0065 | 0.1065 | 0.0860 | 0.0070 | 0.0235 | 0.0040 | 0.0015 | 0.0040 | - | 0.0025 | - |
| 37 | 0.0150 | - | 0.0020 | 0.0040 | 0.0255 | 0.0005 | 0.0230 | 0.0145 | - | 0.0045 | 0.0050 | 0.0010 | 0.0015 | - | 0.0010 | - |
| 38 | 0.0015 | - | 0.0005 | 0.0005 | 0.0060 | 0.0005 | 0.0035 | 0.0025 | - | 0.0010 | 0.0005 | - | 0.0005 | - | - | - |
| 39 | 0.0050 | - | - | 0.0045 | 0.0300 | 0.0025 | 0.0530 | 0.0365 | 0.0025 | 0.0135 | 0.0080 | 0.0030 | 0.0035 | - | 0.0030 | - |
| 40 | 0.0085 | - | 0.0020 | 0.0060 | 0.0530 | 0.0060 | 0.0815 | 0.0825 | 0.0045 | 0.0165 | 0.0095 | 0.0035 | 0.0090 | - | 0.0060 | - |
| 41 | 0.0060 | - | 0.0030 | 0.0110 | 0.1940 | 0.0045 | 0.2100 | 0.1870 | 0.0085 | 0.0250 | 0.0075 | 0.0030 | 0.0035 | - | 0.0015 | - |
| 42 | 0.0030 | - | 0.0020 | 0.0050 | 0.0290 | 0.0015 | 0.0310 | 0.0215 | 0.0015 | 0.0050 | 0.0025 | 0.0010 | 0.0020 | - | 0.0010 | - |
| 43 | 0.0035 | - | 0.0015 | 0.0055 | 0.0610 | 0.0015 | 0.0375 | 0.0310 | 0.0015 | 0.0065 | 0.0030 | 0.0010 | 0.0020 | - | 0.0015 | - |
| 44 | 0.0065 | - | 0.0035 | 0.0080 | 0.0380 | 0.0020 | 0.0615 | 0.0500 | 0.0120 | 0.0115 | 0.0060 | 0.0250 | 0.0045 | - | 0.0030 | - |
| 45 | 0.0035 | - | 0.0035 | 0.0095 | 0.0830 | 0.0045 | 0.0710 | 0.0540 | 0.0020 | 0.0125 | 0.0060 | 0.0025 | 0.0035 | - | 0.0020 | - |
| 46 | 0.0065 | - | 0.0035 | 0.0100 | 0.0485 | 0.0055 | 0.0740 | 0.0550 | 0.1700 | 0.1700 | 0.0120 | 0.0075 | 0.0100 | - | 0.0095 | - |
| **Average** | **0.0087** | **0.0000** | **0.0016** | **0.0061** | **0.0570** | **0.0015** | **0.0371** | **0.0294** | **0.0068** | **0.0113** | **0.0035** | **0.0019** | **0.0021** | **0.0000** | **0.0016** | **0.0** |
| **Maximum** | **0.1150** | **0.0000** | **0.0055** | **0.0275** | **0.3560** | **0.0065** | **0.2100** | **0.1870** | **0.1700** | **0.1700** | **0.0120** | **0.0250** | **0.0100** | **0.0000** | **0.0095** | **0.0** |

- below the LOD (limit of detection)





Figure S1. HPLC-FLD chromatogram of snow
